# A quantitatively-modeled homozygosity mapping algorithm, qHomozygosityMapping, utilizing whole genome single nucleotide polymorphism genotyping data

**DOI:** 10.1186/1471-2105-11-S7-S5

**Published:** 2010-10-15

**Authors:** Huqun*, Shun-ichiro Fukuyama, Hiroyuki Morino, Hiroshi Miyazawa, Tomoaki Tanaka, Tomoko Suzuki, Masakazu Kohda, Hideshi Kawakami, Yasushi Okazaki, Kuniaki Seyama, Koichi Hagiwara

**Affiliations:** 1 Department of Respiratory Medicine, Saitama Medical University, 38 Morohongo, Moroyama, Saitama 350-0495, Japan; 2 Department of Medical Oncology, The Affiliated Hospital of Inner Mongolia Medical College, Tong Dao Bei Jie, 010050 Hohhot, China; 3 Department of Epidemiology, Research Institute for Radiation Biology and Medicine, Hiroshima University, Hiroshima 734-8553, Japan; 4 Division of Functional Genomics and Systems Medicine, Research Center for Genomic Medicine, Saitama Medical University, 1397-1 Yamane, Hidaka City, Saitama 350-1241, Japan; 5 Department of Respiratory Medicine, Juntendo University, School of Medicine, 2-1-1 Hongo, Bunkyo-ku, Tokyo 113-8421, Japan

## Abstract

Homozygosity mapping is a powerful procedure that is capable of detecting recessive disease-causing genes in a few patients from families with a history of inbreeding. We report here a homozygosity mapping algorithm for high-density single nucleotide polymorphism arrays that is able to (i) correct genotyping errors, (ii) search for autozygous segments genome-wide through regions with runs of homozygous SNPs, (iii) check the validity of the inbreeding history, and (iv) calculate the probability of the disease-causing gene being located in the regions identified. The genotyping error correction restored an average of 94.2% of the total length of all regions with run of homozygous SNPs, and 99.9% of the total length of them that were longer than 2 cM. At the end of the analysis, we would know the probability that regions identified contain a disease-causing gene, and we would be able to determine how much effort should be devoted to scrutinizing the regions. We confirmed the power of this algorithm using 6 patients with Siiyama-type α1-antitrypsin deficiency, a rare autosomal recessive disease in Japan. Our procedure will accelerate the identification of disease-causing genes using high-density SNP array data.

## Background

Identification of the genetic factors underlying disease causation provides crucial information for disease prevention and treatment. Nevertheless, genetic factors have not yet been elucidated for many diseases [1,2].

Homozygosity mapping [3] enables the detection of recessive disease-causing genes in a few patients from families with a history of inbreeding; this mapping technique is especially useful for the detection of rare genes. With this technique, chromosomal segments in which all polymorphic markers are homozyogous are considered autozygous segment (AS) [4]. If a patient's coefficient of consanguinity is *F*, and the frequency of the disease-causing gene in the population is *p*, then the chance that the recessive disease-causing gene is located in an AS (*P_AS_*) is.

(1) $$P_{AS}=\frac{F}{(1-F)p+F}$$

[3]

If a patient is from an inbred family (i.e., *F *is large) and the disease is rare (i.e., *p *is small), then *P_AS_*≈ 1, indicating that the gene is located in an AS. There are implementations that utilize single-nucleotide polymorphism (SNP) genotyping data obtained by high-density arrays [5,6]. The usable implementation should (i) correct genotyping errors because thousands of SNPs are mistyped per high-density SNP array, adversely affecting the homozygosity mapping analysis; (ii) search for ASs genome-wide; (iii) check the validity of the inbreeding history, which is vital for homozygosity mapping but is often erroneous, and (iv) calculate the probability of the disease-causing gene being located in the regions identified. At the end of the analysis, we would know the probability that regions identified contain a disease-causing gene, and we would be able to determine how much effort should be devoted to scrutinizing the regions.

In the current study, we present an algorithm that implements the capabilities described in the above paragraph. We confirmed the power of this algorithm using 6 patients with Siiyama-type α1-antitrypsin deficiency, a rare autosomal recessive disease in Japan [7,8]. The preliminary version of the algorithm described here has been used to prove that the *SLC34A2 *gene is responsible for pulmonary alveolar microlithiasis [9]; the current version has been used to show that the *OPTN *gene is responsible for amyotrophic lateral sclerosis [10].

## Implementation

### Crossover model

We used the Haldane's Poisson process model for the occurrence of crossovers and performed all calculations based on this model [11]. Information on SNPs used by Affymetrix's Genome-Wide Human SNP Array 6.0 (hereafter referred to as SNP Array 6.0) was summarized in the annotation file, [12], in which the genetic distance from the telomere of the short arm of a chromosome to each SNP was obtained by interpolation using the sex-averaged data published by deCODE Genetics [13]. We restricted our analysis to a total of 890,625 autosomal SNPs with assigned dbSNP refIDs [14].

### Monte Carlo simulation

**Figure 1:**
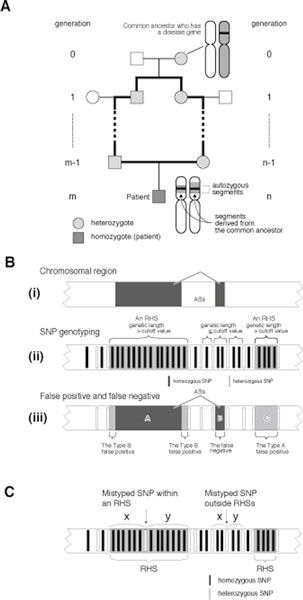
**Connections between AS, RHS, false negative, type A false positive, and type B false positive values**. (A) In a family with a consanguineous marriage, a loop is formed in the pedigree (bold lines). A chromosomal segment that is separately inherited through both sides of the arc becomes homozygous in an offspring and forms an autozygous segment. (B) (i) a chromosomal region with 2 ASs (dark gray boxes). (ii) An RHS is a region whose genetic length greater than the cutoff value. (iii) Relationship of an RHS and an AS. ASs are shown by dark gray boxes, and RHSs are shown by light gray boxes. Three types of errors are defined: false negative, type A false positive, and type B false positive. (C) Principle used for the genotyping error correction. If a homozygous SNP in an RHS is mistyped and becomes heterozygous, it is likely to have a greater distance (i.e. *x *+ *y*) from the adjacent heterozygous SNPs than a heterozygous SNP that exists in another part of the autosomes. Therefore, heterozygous SNPs with a large *x *+*y *are likely to be mistyped.

The average number, the average length, and the maximal length of the ASs derived from a common ancestor were calculated for a range of m + n values (Figure 1A) using a Monte Carlo simulation. The trial was repeated until we observed 100,000 events in which at least 1 AS appeared in the autosomal region.

### The length of AS

The subject is removed from the common ancestor *m *generations on the paternal side and *n *generations on the maternal side (Figure 1A). Assuming that the length of each autosome is infinite, the length of AS conforms to an exponential distribution with a probability density function of

(2) f(x)=λe−λx              λ=m+n100(cM−1).

In actuality, the autosomes have finite length; however, **equation 2 **provides a good approximation when the length of an AS is much shorter than the length of an autosome.

### RHS (run of homozygous SNPs), false negative, type A false positive and type B false positive

An RHS is defined as a run of homozygous SNPs with a genetic length greater than the RHS cutoff value (Figure 1B). All SNPs in an AS are homozygous, and therefore an RHS suggests the presence of an AS. We defined 3 types of errors. False negatives are ASs that are not contained in RHSs. Type A false positives are RHSs that do not contain ASs. Type B false positives are the spaces within an RHS that do not contain an AS. The false negative rate (*R_false negative_*) is the ratio of false negatives to the total length of the AS. The false positive rate (*R*_*false positive*_) is the ratio of false positives (the type A false positives plus the type B false positives) to the total length of the autosomes.

(1) *R_false negative_*, the ratio of the total length of false negatives to the total length of the AS

According to the **equation 2**,

(3) $$\begin{array}{c} R_{false\;negative}=\frac{\int_{0}^{c}xf(x)dx}{\int_{0}^{\infty }xf(x)dx}\\ =1-e^{-\lambda c}(1+\lambda c). \end{array}$$

(2) *R_Type A false positive_*, the ratio of the total length of type A false positives to the total length of the autosomes

Given that *N_SNP _*is the total number of SNPs on a genotyping array, and *P_n _*and *Q_n _*are the frequencies of the major and minor alleles for the nth SNP, respectively, then the average frequencies of the major alleles (${\bar{F}}_{major\;allele}$) and the minor alleles (${\bar{F}}_{minor\;allele}$) are

$${\bar{F}}_{major\;allele}=\frac{\sum_{n=1}^{N_{SNP}}P_{n}}{N_{SNP}}\;\text{and}\;{\bar{F}}_{minor\;allele}=\frac{\sum_{n=1}^{N_{SNP}}Q_{n}}{N_{SNP}},$$

respectively. The numbers of homozygous SNPs (*N_homozygousSNP_*) and heterozygous SNPs (*N_heterozygousSNP_*) are approximated by

$$\begin{array}{l} N_{\mathrm{hom}ozygousSNP}\approx {\left({\bar{F}}_{major\;allele}\right)}^{2}N_{Pt}+{\left({\bar{F}}_{minor\;allele}\right)}^{2}N_{Pt},\;\;\text{and}\\ N_{heterozygousSNP}\approx 2\left({\bar{F}}_{major\;allele}\right)\left({\bar{F}}_{minor\;allele}\right)N_{Pt}, \end{array}$$

where *N_pt _*is the number of SNPs successfully genotyped. Assuming that heterozygous SNPs are randomly located, then the length between 2 heterozygous SNPs conforms to an exponential distribution with a probability density function of

(4) $$\begin{array}{cc} f(x)=\lambda x & \lambda =\frac{N_{heterozygousSNP}}{L_{autosome}}(cM^{-1}) \end{array}\;,$$

where *L_autosome _*is the entire length of the autosomes. Therefore, at a cutoff value of *c *cM,

(5) $$R_{Type\;A\;false\;positive}=\frac{\int_{c}^{\infty }xf(x)dx}{\int_{0}^{\infty }xf(x)dx}=(1+\lambda c)e^{-\lambda c}.$$

(3) *R_Type B false positive_*, the ratio of the total length of type B false positives to the total length of the autosomes

*R_Type B false positive _*is not calculated mathematically but is calculated according to the actual data. An RHS containing an AS is expected to have type B false positives with an average length of $\frac{1}{2}\times \frac{L_{autosome}}{N_{heterozygousSNP}}$ on each end. It is impossible to distinguish RHSs that contain ASs from those that do not. We calculated *R_Type B false positive _*under the assumption that every RHS contains an AS. Therefore, the *R_Type B false positive _*calculation results in an overestimation, which we consider better than an underestimation for determination of the appropriate RHS cutoff. Therefore,

(6) $$R_{Type\;B\;false\;positive}=\frac{number\;of\;RHS}{2}\times \frac{L_{autosome}}{N_{heterozygousSNP}}.$$

(4) *R_false positive_*, the ratio of the total length of false positives to the total length of the autosomes

(7) $$\begin{array}{c} R_{false\;positive}=R_{Type\;A\;false\;positive}+R_{Type\;B\;false\;positive}\\ =(1+\frac{N_{heterozygousSNP}}{L_{autosome}}c)e^{-\frac{N_{heterozygousSNP}}{L_{autosome}}c}+\frac{number\;of\;RHS}{2}\times \frac{L_{autosome}}{N_{heterozygousSNP}}. \end{array}$$

### Probability that a disease-causing gene is contained in RHSs, or the overlap of RHSs

The probability that RHSs obtained contains a disease-causing gene is calculated using **equation 1**.

(8) $$\begin{array}{c} P_{GeneIsInRHS}=(1-R_{false\;negative})\times P_{AS}\\ =(1-R_{false\;negative})\times \frac{F}{(1-F)p+F}. \end{array}$$

Here, *F *is the coefficient of consanguinity and is calculated by

(9) $$F\approx \frac{\text{total length of RHSs}}{\text{total length of the autosomes}}.$$

The probability that the overlap of RHSs among multiple patients contain the gene is calculated by

(10) $$P_{GeneIsInOverlap}=\prod_{All\;patients}P_{GeneIsInRHS}.$$

### Human Subjects and genotyping

This study was approved by the Institutional Review Boards of Saitama Medical University and Juntendo University. After obtaining written informed consent, DNA samples from 6 patients with α1-antitrypsin deficiency were purified from peripheral blood. These patients were not related and lived in different areas of Japan. Patients 1-5 were from families with a history of inbreeding because their parents were first cousins. Patient 6 did not have any family history of inbreeding. These 6 patients were genotyped using the SNP Array 6.0. The genotyping data for 86 HapMap JPT were available in the HapMap3 draft release 2 http://www.hapmap.org, and were downloaded from the Wellcome Trust Sanger Institute web site http://www.sanger.ac.uk/humgen/hapmap3/. The genotyping data for NA18987, a subject in HapMap JPT, was also distributed from Affymetrix and was used in the current study.

### Genotyping error correction

Genotyping errors may convert homozygous SNPs to heterozygous SNPs and erroneously terminate an RHS, resulting in the failure to detect a portion of an RHS. According to Affymetrix, SNP Array 6.0 has an accuracy of > 0.997, implying that the genotyping error rate (*P_genotypingError_*) may be 0.003 at maximum. A mistyped heterozygous SNP occurring in an RHS is separated by a large distance from neighboring heterozygous SNPs (Figure 1C). Therefore, if a heterozygous SNP is separated from neighboring SNPs by a distance that is rarely observed by chance, we speculated that the SNP was mistyped. Using **equation 4**, we calculated the probability of a heterozygous SNP being separated from neighboring SNPs at the observed distance (*P_distanceOccurredByChance_*). A SNP with *P_distanceOccreceByChance _*< 0.01 was considered a mistyped SNP and these data were removed. This algorithm may erroneously remove 20 correctly genotyped heterozygous SNPs (*N_homozygousSNP _*x *P_genotypingError _*x 0.01) from a single SNP array analysis data, which we considered acceptable.

### Statistical analysis

The number of patients and controls who shared an RHS at each SNP position was compared. The assumption was made that

$$u=\frac{{\overset{\wedge }{p}}_{1}^{*}-{\overset{\wedge }{p}}_{2}^{*}}{\sqrt{\overset{\wedge }{p}*(1-{\hat{p}}^{*})\left(\frac{1}{n_{1}}+\frac{1}{n_{1}}\right)}}$$

has a standard normal distribution, where ${\hat{p}}_{1}{}^{*}=\frac{x_{1}+0.5}{n_{1}+1}$, ${\hat{p}}_{2}{}^{*}=\frac{x_{2}+0.5}{n_{2}+1}$, ${\hat{p}}^{*}=\frac{x_{1}+x_{2}+0.5}{n_{1}+n_{2}+1}$. Here, *x*_1 _and *x*_2 _represent the numbers of patients and controls sharing RHSs, respectively, and *n*_1 _and *n*_2 _represent the total numbers of patients and controls, respectively. The *P *value was calculated by

$$P=\int_{u_{0}}^{\infty }\frac{1}{\sqrt{2\pi }}e^{-\frac{x^{2}}{2}}dx.$$

### Computer program

The computer program was written in the ANSI standard C programming language. The program was compiled by the GNU C compiler 4.2 and run on a MacBook Pro (CPU: 2.53 GHz Intel Core 2 Duo, 4 GB RAM) computer. The command line programs and the programs equipped by graphic user interface are both available from our web site at http://www.hhanalysis.com.

## Result

### Strategy

Our aim was to establish an algorithm for homozygosity mapping that uses SNP genotyping data obtained by high-density arrays, is equipped by a powerful genotyping error correction algorithm, detects ASs genome-wide, allows investigation into the family inbreeding history, and is able to calculate the probability that the identified regions contain the target gene.

The algorithm searches for the ASs (Figure 1A, B(i)) through runs of homozygous SNPs, or RHSs, that are formed by consecutively homozygous SNPs and are longer than the RHS cutoff value (Figure 1B(ii)). RHSs are presumably the autozygous segments (ASs). Three types of errors were defined; false negative, type A false positive, and type B false positive (Figure 1B(iii)). The main determinants of the false negative rate (*R_false negative_*), which is the ratio of the total length of false negatives to the total length of ASs, are the number of SNPs investigated and the genotyping error rate. The main determinants of the false positive rate (*R_false positive_*), which is the ratio of the total length of type A false positives plus type B false positives to the entire length of the autosomes, are the positioning of SNPs, local haplotype block structure [15], and population substructure [16].

To attain the aims stated above while avoiding the influence of these errors, our algorithm had the following steps: **Step **(**a**) determine an appropriate RHS cutoff value based on the Haldane's recombination model; **Step **(**b**) perform genotyping error correction; **Step **(**c**) detect RHSs; **Step **(**d**) obtain the overlaps of RHSs among patients; and **Step **(**e**) correct false positives by a case-control approach. The validity of the family history is checked at **Step (c)**. We used 5 patients with Siiyama-type α1-antitrypsin deficiency, a rare disease in Japan, to verify our strategy. Analyses performed in the Result section can be reproduced using the program contained in additional file 1 according to the tutorial also contained in the additional file 1.

### Determination of the RHS cutoff

**Figure 2:**
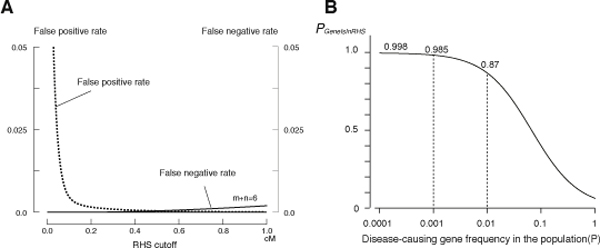
**Determination of the RHS cutoff and the probability that the disease-causing gene is contained in RHSs**. (A) The false negative rate (*R_false negative_*) and the false positive rate (*R_false positive_*) were calculated using **equations 3 **and **7 **using the genotyping data for 5 α1-antitrypsin deficiency patients. The false negative rate shown is for a child from a first-cousin marriage (m + n = 6). (B) The probability that RHSs contain the disease gene (*P_GeneIsInRHS_*) calculated for a child from a first-cousin marriage. The coefficient of consanguinity (*F*) used was 1/16, which was calculated according to Wright {Wright, S. Systems of Mating. V. General Considerations *Genetics* 1921: **6**:167-178}. *F *can be more precisely calculated as the total length of RHSs divided by the total length of the autosomes for the actual calculation (**equation 9**). *P_GeneIsInRHS _*varies depending on the frequency of the gene in the population.

The expected false negative and false positive rates for the SNP Array 6.0 from the Haldane's model were calculated by using **equation 3 **and **7 **[**Step (a)**] (Figure 2A). We gave the priority to reducing the false positive rate than to reducing the false negative rate, because we empirically determined that it simplified the analysis. We chose 0.6 cM as the RHS cutoff value, at which the false negative rate was 0.0006 and the false positive rate was 0.0029. The probability that the RHSs contained the disease-causing gene (*P_GeneIsInRHS_*) at this condition was calculated using **equation 8 **(Figure 2B).

### Genotyping error correction

**Figure 3:**
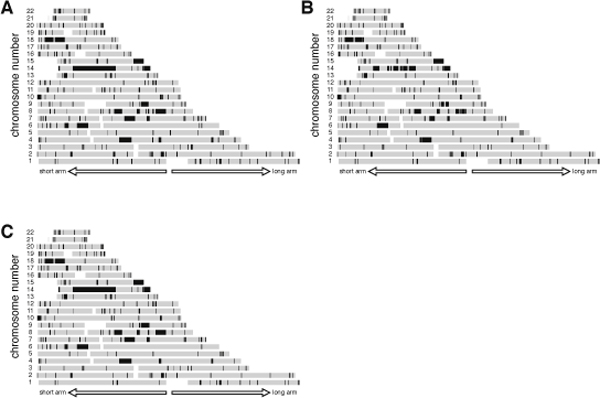
**Genotyping error corrections**. (A) RHSs for NA18987. (B) RHSs detected after introducing genotyping errors to 2,105 SNPs. (C) RHSs after the genotyping error correction algorithm was applied.

The power of the genotyping error correction algorithm was investigated using genotyping data for subject NA18987 (female) from HapMap JPT. The subject was independently genotyped in HapMap draft 3 and by Affymetrix, and data were made public from both sources. A comparison of these 2 datasets revealed that the genotyping results for 701,753 SNPs matched between these 2 sources, and they were therefore considered highly accurate. Using the matched data, RHSs were obtained with an RHS cutoff value of 0.6 cM (Figure 3A). The presence of a long RHS (36.2 cM at maximum) suggested that she had a family history of inbreeding, as described later. Considering the fact that the manufacturer (Affymetrix) claimed that the genotyping error rate for the SNP Array 6.0 is less than 0.003, we randomly introduced errors into selected 2,105 SNPs (701,753 SNPs × 0.003) and obtained RHSs. These error hampered the detection of RHSs, especially the long ones (Figure 3B). Following application of the genotyping error correction algorithm (Figure 1C), RHSs were restored (Figure 3C). The same trial repeated 100 times revealed that the genotyping error correction restored an average of 94.2% of the total length of all RHSs, and 99.9% of the total length of RHSs that were longer than 2 cM. This indicated that 99.9% of the total length of ASs resulting from first- or second cousin marriages would be correctly detected as RHSs after the correction. The total length of the regions that were erroneously detected as RHSs amounted to only 0.2% of the total length of the autosomes. These results indicated that the performance of the genotyping error correction algorithm was excellent.

### RHSs in the patients

**Figure 4:**
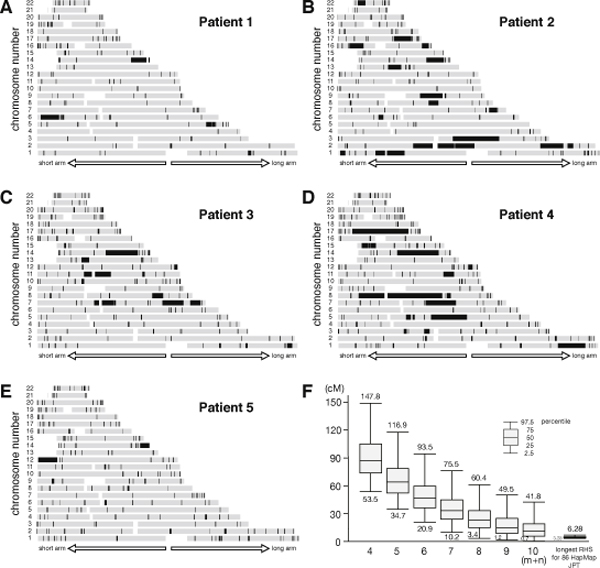
**RHSs obtained for 5 patients with Siiyama-type α1-antitrypsin deficiency and the distribution of the longest AS obtained by a Monte Carlo simulation**. (A) - (E) RHSs for each patient. (F) The distribution of the length of the longest AS obtained by a Monte Carlo Simulation. The distribution for 86 HapMap JPT patients is also shown in the right side.

We applied the genotyping error correction algorithm to the data for 5 patients with Siiyama-type α1-antitrypsin deficiency **[Step (b)]**, and then obtained RHSs [**Step (c)**] (Figure 4A-E). All patients had long RHSs, which were likely to be the result of first-cousin marriages.

### Statistics of AS

We investigated whether the RHSs obtained for each patient were consistent with family history [**Step (d)**]. We focused on the size of the longest AS because they are an index of the most recent occurrence of inbreeding in the patient's family (**equation 2**). The distribution of the length of the longest AS is calculated by a Monte Carlo simulation (Figure 4F). From this distribution we are able to say that the family history of a first cousin marriage (m + n = 6) is unlikely when the longest RHS is less than 20.9 cM. The size of the longest RHS for Patients 1-5 were consistent with what expected from their family histories (Table 1).

**Table 1:** Size of the longest RHS for each patient

	Length of the longest RHS (cM)
Patient 1	36.2
Patient 2	39.6
Patient 3	22.1
Patient 4	40.3
Patient 5	30.2

### Overlap of RHSs

**Figure 5:**
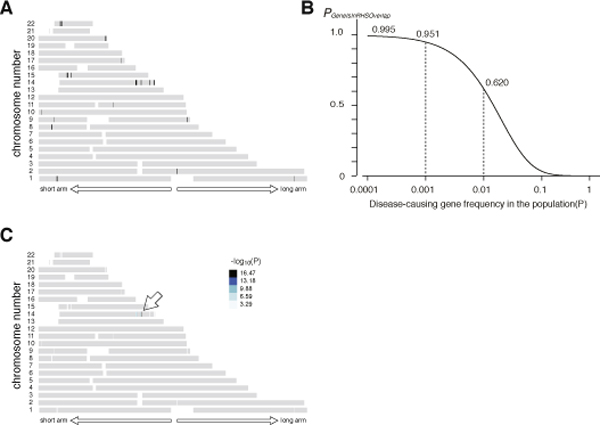
**Case-control analysis**. (A) The overlaps of RHSs for Patients 1-5. (B) The probability that the disease-causing gene is contained in the overlap (*P_GeneIsInRhsOverlap_*). The probability was calculated by multiplying *P_GeneIsInRHS _*for Patients 1-5. *F *for each patient was calculated as the total length of RHSs divided by the total length of the autosomes. (C) -log_10_(P) value obtained by a case-control analysis. The region pointed by an arrow attained the maximal value 16.47.

We then obtained the overlaps of the RHSs for Patients 1-5 whose parents were first cousins [**Step (d)**] (Figure 5A). The probability that these regions contained the disease-causing gene (*P*_*GeneIsInOverlap*_) was calculated by **equation 10 **and is shown in Figure 5B. The prevalence of Siiyama-type α1-antitrypsin deficiency is less than 1 in a million in Japan, and the frequency of the gene is suspected to be less than 0.001 in the general population, indicating that the overlaps likely contained the disease-causing gene.

Some of the autosomal regions are prone to type A or type B false positives, and thus are likely to appear as an overlap [**Step (e)**]. To prioritize regions for in-depth analysis, we performed a case-control study using 86 HapMap JPT subjects as controls. One overlap had the largest -log_10_(P) value (16.47) and was considered to be the candidate region (Figure 5C). This region (between rs10134551 and rs910349) had a genetic length of 1.44 cM, and contained 15 genes (Table 2), one of which was the disease-causing gene for Siiyama-type α1-antitrypsin deficiency, SERPIN1.

**Table 2:** Genes present in the candidate RHS overlap

*C14orf48*	chromosome 14 open reading frame 48
*OTUB2*	OTU domain, ubiquitin aldehyde binding 2
*DDX24*	DEAD (Asp-Glu-Ala-Asp) box polypeptide 24
*IFI27L1*	interferon, alpha-inducible protein 27-like 1
*IFI27*	interferon, alpha-inducible protein 27
*IFI27L2*	interferon, alpha-inducible protein 27-like 2
*PPP4R4*	protein phosphatase 4, regulatory subunit 4
*SERPINA10*	serpin peptidase inhibitor, clade A (alpha-1 antiproteinase, antitrypsin), member 10
*SERPINA6*	serpin peptidase inhibitor, clade A (alpha-1 antiproteinase, antitrypsin), member 6
*LOC10028*	Description: hypothetical protein LOC100287997
*SERPINA2*	serpin peptidase inhibitor, clade A (alpha-1 antiproteinase, antitrypsin), member 2
*SERPINA1*	serpin peptidase inhibitor, clade A (alpha-1 antiproteinase, antitrypsin), member 1
*SERPINA11*	serpin peptidase inhibitor, clade A (alpha-1 antiproteinase, antitrypsin), member 11
*SERPINA9*	serpin peptidase inhibitor, clade A (alpha-1 antiproteinase, antitrypsin), member 9
*SERPINA12*	serpin peptidase inhibitor, clade A (alpha-1 antiproteinase, antitrypsin), member 12

### A patient without family history of inbreeding

**Figure 6:**
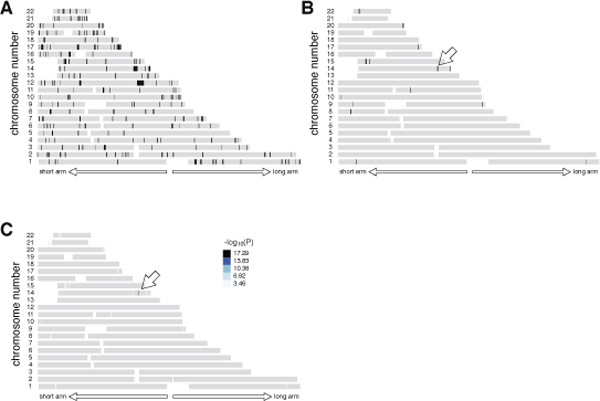
**Subject without family history of inbreeding**. (A) RHSs obtained for a patient without a family history of inbreeding (Patient 6). (B) RHS overlaps for Patients 1-6. Addition of data for Patient 6 further narrowed the overlapped regions (compare with **Figure 5**). The disease-causing gene was contained in the region indicated by a white arrow. (C) -log_10_(P) value obtained by a case-control analysis. The region pointed by an arrow attained the maximal value 17.29.

We occasionally encounter patients who do not have a family history of inbreeding while searching for a recessive disease-causing gene. Data from such patients are not used in the main analysis, but these data may be used for prioritizing the overlaps of RHSs as obtained in Figure 5 for an in-depth search. Patient 6 had Siiyama-type α1-antitrypsin deficiency but did not have a family history of inbreeding. The length of the longest RHS (6.8 cM, Figure 6A) was outside of the 95% range for the Japanese population (Figure 4F, **rightmost bar and whisker**). We reasoned that the patient's family might have had forgotten inbreeding history, and that the RHSs for the patient may have a high probability of containing the disease-causing gene. This was indeed the case; addition of the data from Patient 6 excluded several overlapped regions (Figure 6B, **compare with **Figure 5A) and increased -log_10_(P) (Figure 6C, **compare with **Figure 5C), although the list of the genes was the same as Table 2. If the length of the longest RHS suggested a hidden inbreeding history, the data for subjects without an inbreeding history could be used to prioritize some RHS overlaps for an in-depth search.

## Discussion

In the current report, we described the quantitatively-modeled homozygosity mapping algorithm that uses high density array SNP genotyping data.

Homozygosity mapping is simple in principle, but many pitfalls were discovered when it was actually applied. Problems that included (i) unexpected allelic heterogeneity, (ii) identification of a homozygous identical-by-descent (IBD) region to the disease locus, (iii) underestimation of the extent of inbreeding, were pointed out in the analyses using microsatellite markers [17] and are still observed in the analyses using SNPs. Moreover, use of high-density SNP arrays introduced a novel problem, (iv) a large number of mistyped SNPs. Although the genotyping error rate is low for high-density arrays, the huge number of SNPs in these arrays inevitably produces a large number of mistyped SNPs. Even a single mistyped SNP erroneously terminates an RHS, making the detection of large RHSs difficult. Our algorithm has overcome all these problems: problem (i) is solved by using high-density SNP arrays, problem (ii) by case-control approach, problem (iii) by identifying ASs as RHSs and calculating *F *by the total length of RHSs divided by the total length of the autosomes, and problem (iv) by applying genotyping error correction algorithm.

As stated as Problem (ii) above, we observed some autosomal regions had a high probability of having RHSs. This may be caused by SNP positioning, local haplotype block structure, or population substructure. The effect of them was eliminated by using a case-control approach, which is performed in the order that (a) obtain overlap of RHS among patients, and (b) perform a case-control analysis targeting obtained overlaps.

Homozygosity mapping has power to identify a disease-causing gene in as few as 3 patients, and we have indeed identified the *SLC34A2 *gene in pulmonary alveolar microlithiasis and the *OPTN *gene in the amyotrophic lateral sclerosis both in 3 patients [9,10]. Amyotrophic lateral sclerosis has multiple causative genes. In the latter report, we were able to identify one of the genes by investigating each combination of 3 patients from 7 patients with a history of inbreeding, seeking for 3 patients harboring the same disease-causing gene. Our algorithm worked fine in this approach. During the process, it was quite helpful that the algorithm provided the probability that the identified regions contain the disease-causing gene, which determined how much effort should be further devoted. To our knowledge, the algorithm presented in the current study is the first to provide this information.

## Conclusions

We described an algorithm that enables homozygosity mapping to be performed based on a quantitative model using SNP genotyping data. Our procedure will accelerate the identification of disease-causing genes using high-density SNP array data.

## Availability and requirements

Project name: qHomozygosityMapping

Project home page: http://www.hhanalysis.com

Operating system(s): Mac, Linux and Windows.

Programming language: C

License: GNU GPL.

Any restrictions to use by non-academics: The software is for academic purpose only.

## Funding

This work is supported in part by the grant-in-aid for scientific research (No. 18390242) from the Japan Society of Promotion of Science, and in part by the grants-in-aid for Health and Labor Science (Nos. H22-Nanchi-Ippan-005 and H20-Nanchi-Ippan-023) from the Ministry of Health, labor and Welfare, Japan.

## Competing interests

The authors declare that they have no competing interests.

## Authors' contribution

Huqun, S.F., H.M., T.T., T.S., M.K., H.K., Y.O., and K.S. tested the programs, did genetic analyses and provided ideas to improve the program. K.S. collected the patients' samples. K.H. provided basic ideas, wrote the program, and prepared manuscript.

## Supplementary Material

Additional file 1 **This file is a zipped package that contains programs and tutorial for Linux, MacOS X and Windows platforms**. Click here for file
