# Supplementary material for: A quantitatively-modeled homozygosity mapping algorithm, qHomozygosityMapping, utilizing whole genome single nucleotide polymorphism genotyping data
Source: BMC Bioinformatics. 2010 Oct 15;11(Suppl 7):S5. doi: 10.1186/1471-2105-11-S7-S5 (PMC2957688; doi:10.1186/1471-2105-11-S7-S5)
Supplement: Additional file 1 — This file is a zipped package that contains programs and tutorial for Linux, MacOS X and Windows platforms. [file 1471-2105-11-S7-S5-S1.zip › supplementary file/TutorialAndProgram/HMmanualLinux.doc]

**Tutorial**

**(I) Preparation**

(A)	Softwares

You need a viewer for the graphic files written in the PostScript programming language. The program described below works as a PostScript viewer. Many of the Linux distribution has a PostScript viewer built-in.

Document Viewer	http://www.gnome.org/projects/evince

GhostScript and GSView	http://pages.cs.wisc.edu/~ghost/

(B)	Directories and files

1.	Copy the content of “Linux_i386” directory to your desktop. A total of 8 directories will be copied.

***Please note that you need to change the name “myhomedirectory” to the name of your home directory.***

```
/TutorialAndProgram/Linux_i386/HMAnalysisProgramDirectory
/TutorialAndProgram/Linux_i386/AnnotationFileDirectory
/TutorialAndProgram/Linux_i386/AnalysisFileDirectory
/TutorialAndProgram/Linux_i386/CaseDirectory
/TutorialAndProgram/Linux_i386/ControlDirectory
/TutorialAndProgram/Linux_i386/ResultDirectory
/TutorialAndProgram/Linux_i386/TargetAreaFileDirectory
/TutorialAndProgram/Linux_i386/ExcludedAreaFileDirectory

	-> /home/myhomedirectory/Desktop/HMAnalysisProgramDirectory
	-> /home/myhomedirectory/Desktop/AnnotationFileDirectory
	-> /home/myhomedirectory/Desktop/AnalysisFileDirectory
	-> /home/myhomedirectory/Desktop/CaseDirectory
	-> /home/myhomedirectory/Desktop/ControlDirectory
	-> /home/myhomedirectory/Desktop/ResultDirectory
	-> /home/myhomedirectory/Desktop/TargetAreaFileDirectory
	-> /home/myhomedirectory/Desktop/ExcludedAreaFileDirectory
```

Directories copied to the Desktop

2.	Copy the annotation file for Japanese from “/ TutorialAndProgram/AnnotationFiles” directory to “AnnotationFileDirectory”.

See appendix for the format of the annotation file.

```
/TutorialAndProgram/AnnotationFiles/annotationJapanese1000k.txt
   -> /home/myhomedirectory/Desktop/AnnotationFileDirectory/annotationJapanese1000k.txt
```

3.	Copy the genotyping data from “TutorialAndProgram/SampleDataSet/A1AT1000kSynthetic” directory into “/home/myhomedirectory/Desktop/CaseDirectory” and from “TutorialAndProgram/SampleDataSet/HapMap3JPNSynthetic” to “/home/myhomedirectory/Desktop/ControlDirectory”.

These genotyping data are artificially made utilizing the data for α1-antitrypsin deficiency patients and HapMap3 subjects, and provide RHSs at the same positions as the original data. This enables to distribute sample datasets without distributing the original data.

See appendix for the format of genotyping data.

```
/TutorialAndProgram/SampleDataSets/A1AT1000kSynthetic/*.*
                                  -> /home/myhomedirectory/Desktop/CaseDirectory/*.*

/TutorialAndProgram/SampleDataSets/HapMap3JPNSynthetic/*.*
                                  -> /home/myhomedirectory/Desktop/ControlDirectory/*.*
```

**(II)	RHS cutoff determination**

(A)	Analysis file

Copy “/TutorialAndProgram/SampleAnalysisFile/Linux/RHSCutoffDetermination/ RHSCutoffAnalysisFile.txt” to AnalysisFileDirectory.

```
/TutorialAndProgram/SampleAnalysisFile/Linux/RHSCutoffDetermination/RHSCutoffDetermination.txt
   -> /home/myhomedirectory/Desktop/AnalysisFileDirectory/RHSCutoffAnalysisFile.txt
```

Open the file. **Change “myhomedirectory” to the name of your home directory.**

::patient pool

/home/myhomedirectory/Desktop/CaseDirectory/01.txt

/home/myhomedirectory/Desktop/CaseDirectory/02.txt

/home/myhomedirectory/Desktop/CaseDirectory/03.txt

/home/myhomedirectory/Desktop/CaseDirectory/04.txt

/home/myhomedirectory/Desktop/CaseDirectory/05.txt

/home/myhomedirectory/Desktop/CaseDirectory/06.txt

::annotation file

/home/myhomedirectory/Desktop/AnnotationFileDirectory/annotationJapanese1000k.txt

::output directory

/home/myhomedirectory/Desktop/ResultDirectory

RHSCutoffAnalysisFile.txt

(B)	Launch the program

Launch “Terminal”. From inside the terminal, launch “RHSDeterminationProg” using the analysis file as an augment. You can construct the command line by drag-and-droping “RHSDeterminationProg” and “RHSCutoffAnalysisFile.txt” to the terminal window.

```
>/home/myhomedirectory/Desktop/HMAnalysisProgramDirectory/RHSCutoffDeterminationProg
  /home/myhomedirectory/AnalysisFileDirectory/RHSCutoffAnalysisFile.txt
```

If you cannot launch program because of “permission denied” message, change the permission of the program using chmod.

chmod +x /home/myhomedirectory/Desktop/HMAnalysisProgramDirectory/RHSCutoffDeterminationProg

(C)	Check the result

The program outputs a message that shows RHS cutoff, the rates of type A false positive, type B false positive, false negative for m + n = 6, and false negative for m + n = 10.

```
		RHScutoff	TypeAFP	TypeB_FP	FN_m+n=6	FN_m+m=10
OutputData01	0.05	0.14390	0.04336	0.00000	0.00001
OutputData02	0.10	0.01620	0.02557	0.00002	0.00005
OutputData03	0.15	0.00274	0.01794	0.00004	0.00011
```

…

The program also outputs a graph file named “GraphOfErrors.ps” that is similar to what shown in Figure 2A in the manuscript.

GraphOfErrors.ps

**(III)	SingleGroup analysis to obtain RHSs for each subject and overlaps of RHSs for multiple subjects**

(A)	Analysis file

Copy “/TutorialAndProgram/SampleAnalysisFile/Linux/SingleGroupAnalysis/ SingleGroupAnalysisFile.txt” to “AnalysisFileDirectory”.

```
/TutorialAndProgram/SampleAnalysisFile/Linux/SingleGroupAnalysis/SingleGroupAnalysisFile.txt
   -> /home/myhomedirectory/Desktop/AnalysisFileDirectory/SingleGroupAnalysisFile.txt
```

Open the file. **Change “myhomedirectory” to the name of your home directory.**

::patient pool

/home/myhomedirectory/Desktop/CaseDirectory/01.txt

/home/myhomedirectory/Desktop/CaseDirectory/02.txt

/home/myhomedirectory/Desktop/CaseDirectory/03.txt

/home/myhomedirectory/Desktop/CaseDirectory/04.txt

/home/myhomedirectory/Desktop/CaseDirectory/05.txt

/home/myhomedirectory/Desktop/CaseDirectory/06.txt

::control pool

::excluded area file

::target area file

::annotation file

/home/myhomedirectory/Desktop/AnnotationFileDirectory/annotationJapanese1000k.txt

::output directory

/home/myhomedirectory/Desktop/ResultDirectory

::suspected genotyping error rate

0.003

SingleGroupAnalysisFile.txt”

We use genotyping error correction algorithm (Figure 3 in the manuscript).

(B)	Launch the program

Launch “Terminal” that is stored in the “Application/Utilities”. Launch “HMAnalysisProg” from inside “Terminal” using the analysis file as an augment. You can construct the command line by drag-and-droping “HMAnalysisProg” and “HMAnalsyisAnalysisFile.txt” to the terminal window.

```
>/home/myhomedirectory/Desktop/HMAnalysisProgramDirectory/HMAnalysisProg
  /home/myhomedirectory/Desktop/AnalysisFileDirectory/SingleGroupAnalysisFile.txt
```

If you cannot launch program because of “permission denied” message, change the permission of the program using chmod.

chmod +x /home/myhomedirectory/Desktop/HMAnalysisProgramDirectory/HMAnalysisProg

The program asks you the RHS cutoff value. Type 0.6.

The program output a message. Hit 1 2 and return. The program again output a message. Hit 1 2 3 4 5 and return. Then just hit return and the program terminates.

Input the numbers for the subjects among whom you wish to obtain the RHSs (just hit return to exit) 1 2

Input the numbers for the subjects among whom you wish to obtain the RHSs (just hit return to exit) 1 2 3 4 5

Input the numbers for the subjects among whom you wish to obtain the RHSs (just hit return to exit)

Completed!

(C)	Result

At this point, there are many files output into “ResultDirectory”. The description of each file is provided below.

1a.	HM_001_RHSdata_PatientPool.ps

A PostScript file illustrating RHSs for patient 1 that is presented in Figure 4A.

HM_001_RHSdata_PatientPool.ps

1b.	HM_001_RHSdata_PatientPool.txt

A text file that lists the start and the end of each RHS.

```
1	5883090	9.861487	rs2312464	start
1	6075993	10.561376	rs3789530	end
1	6140446	10.944041	rs9434741	start
1	6489836	11.681913	rs4908553	end
1	16676928	29.938894	rs156847	start
1	17171083	30.819518	rs7513616	end
1	59115556	83.230238	rs2716129	start
1	59342417	83.870053	rs3010325	end
……
```

The columns indicate chromosome number, nucleotide position, genetic map position in centimorgans from the terminal of the short arm of the chromosome, dbSNP rsID, and the label specifying the start and the end of each RHS.

2	Probability_GeneIsInRegionFor_St001.ps

Probability that the target gene is contained in the RHSs for subject 1 similar to what is shown in Figure 2B.

Probability_GeneIsInRegionFor_St001.ps

3a.	HM_004_FoldOverlaps_PatientPool.ps

Autosomal regions in which 4 or more subjects share RHSs.

HM_004_FoldOverlaps_PatientPool.ps

3b.	HM_004_FoldOverlaps_PatientPool.txt

A text file that lists the start and the end of each RHS.

```
1	10985630	18.376669	rs11121676	start	Subjects	 02.txt 03.txt 05.txt 06.txt
1	11306426	19.514301	rs4845992	end	Subjects	 02.txt 03.txt 05.txt 06.txt
1	16676928	29.938894	rs156847	start	Subjects	 01.txt 02.txt 03.txt 04.txt 05.txt
1	17180745	30.836736	rs9435732	end	Subjects	 02.txt 03.txt 04.txt 05.txt
1	26815924	45.655625	rs6673174	start	Subjects	 02.txt 03.txt 04.txt 05.txt
1	27250899	46.334886	rs17162431	end	Subjects	 02.txt 03.txt 04.txt 05.txt
……
```

The columns indicate chromosome number, nucleotide position, genetic map position in centimorgans from the terminal of the short arm of the chromosome, dbSNP rsID, label indicating the start and the end of each RHS, and the list of the subjects (shown as file names) involved.

4a.	HM_NoStSharingRHSs_PatientPool.ps

Number of subjects who share RHSs.

HM_NoStSharingRHSs_PatientPool.ps

4b.	HM_NoStSharingRHSs_PatientPool.txt

A text file that lists the start and the end of the regions according to the number of subjects sharing RHS.

```
1	2715812	3.038907	rs7538063	1	start
1	4405826	6.202897	rs780587	1	end
1	4406705	6.205227	rs10799233	2	start
1	4653917	6.933297	rs7525374	2	end
1	4657092	6.943136	rs7549821	1	start
1	5880176	9.855291	rs7519953	1	end
…
```

The columns indicate chromosome number, nucleotide position, genetic map position in centimorgans from the terminal of the short arm of the chromosome, dbSNP rsID, number of subjects sharing RHS, and the label indicating the start and the end of each RHS.

5a.	HM_RHSsSharedBy002_st001_st002.ps

Overlap of RHSs for patient 1 and patient 2.

HM_RHSsSharedBy002_st001_st002.ps

5b.	HM_RHSsSharedBy002_st001_st002.txt

A text file that lists the start and the end of each RHS.

```
1	5883090	9.861487	rs2312464	start	Subjects	 01.txt 02.txt
1	6075993	10.561376	rs3789530	end	Subjects	 01.txt 02.txt
1	6140446	10.944041	rs9434741	start	Subjects	 01.txt 02.txt
1	6489836	11.681913	rs4908553	end	Subjects	 01.txt 02.txt
1	16676928	29.938894	rs156847	start	Subjects	 01.txt 02.txt
1	17171083	30.819518	rs7513616	end	Subjects	 01.txt 02.txt
……
```

The columns indicate the chromosome number, nucleotide position, genetic map position in centimorgans from the terminal of the short arm of the chromosome, dbSNP rsID, label indicating the start and the end of each RHS, and the list of the subjects (shown as file names) involved.

5c.	Probability_GeneIsInRegionFor002_st001_st002.ps

Graph for the probability that the target gene is contained in the overlap of RHSs for subject 1 and 2.

Probability_GeneIsInRegionFor002_st001_st002.ps

6a.	HM_RHSsSharedBy005_st001_st002_st003_st004_st005.ps

Overlap of RHSs for patient 1 through 5 as shown in Figure 5a.

HM_RHSsSharedBy005_st001_st002_st003_st004_st005.ps

6b.	HM_RHSsSharedBy005_st001_st002_st003_st004_st005.txt

A text file that lists the start and the end of each RHS.

```
1	16676928	29.938894	rs156847	start	Subjects	 01.txt 02.txt 03.txt 04.txt 05.txt
1	17171083	30.819518	rs7513616	end	Subjects	 01.txt 02.txt 03.txt 04.txt 05.txt
1	233182132	242.831039	rs6586357	start	Subjects	 01.txt 02.txt 03.txt 04.txt 05.txt
1	233309887	243.461447	rs11580121	end	Subjects	 01.txt 02.txt 03.txt 04.txt 05.txt
2	126453217	138.137384	rs10496652	start	Subjects	 01.txt 02.txt 03.txt 04.txt 05.txt
2	126744948	138.587802	rs193332	end	Subjects	 01.txt 02.txt 03.txt 04.txt 05.txt
……
```

The columns indicate chromosome number, nucleotide position, genetic map position in centimorgans from the terminal of the short arm of the chromosome, dbSNP rsID, label indicating the start and the end of each RHS, and the list of the subjects (shown by file names) involved.

6c.	Probability_GeneIsInRegionFor005_st001_st002_st003_st004_st005.ps

Probability that the target gene is contained in the overlap of RHSs for subject 1 through 5.

Probability_GeneIsInRegionFor005_st001_st002_st003_st004_st005.ps

7.	HM_HomozygosityMapping_Summary_PatientPool.txt

A text file that summarizes the genotype for all subjects.

```
chr	pos	gen map	rsID	'A' frequency
1	554484	0.620449	rs10458597	0.977800
1	554636	0.620619	rs9629043	0.000000
1	557616	0.623954	rs11510103	0.033300
1	711153	0.795757	rs12565286	0.855600
1	730720	0.817652	rs12082473	0.855600

01.txt	02.txt	03.txt	04.txt	05.txt	06.txt	No.
BB	BB	BB	BB	BB	BB	0
AA	AA	AA	AA	AA	AA	1
BB	BB	BB	BB	BB	BB	2
BB	BB	BB	BB	BB	BB	3
BB	BB	BB	BB	BB	BB	4
```

8.	HM_FoldOverlap_Summary_PatientPool.txt

A text file that summarizes the position of RHSs for all subjects.

```
chr	pos	gen map	rsID	'A' frequency
14	84759628	83.623609	rs2767538	0.733300
14	84763227	83.625376	rs1667516	0.033300
14	84763606	83.625562	rs11159695	0.622200
14	84763657	83.625588	rs1667515	0.633300
14	84764125	83.625817	rs1667514	1.000000
14	84764638	83.626069	rs12433588	1.000000

01.txt	02.txt	03.txt	04.txt	05.txt	06.txt	overlaps	No.
0	1	1	1	0	0	3	719232
0	1	1	1	0	0	3	719233
0	1	1	1	0	0	3	719234
0	1	1	1	0	0	3	719235
0	1	1	1	0	0	3	719236
0	1	1	1	0	0	3	719237
```

The columns indicate chromosome number, nucleotide position, genetic map position in centimorgans from the terminal of the short arm of the chromosome, dbSNP rsID, frequency of the “A” allele, a mark whether each subject has an RHS at the position (1, RHS(+); 0, RHS(-) ), the number of subjects who has an RHS at the position, and the serial number of the SNP starting from the 1st SNP (the SNP located nearest to the p-terminal of chromosome 1) used in this analysis.

**(IV)	Case-control analysis to obtain RHS overlap that is specific to case**

Some regions of the autosomes become homozygous more frequently than the other part. We need to find out RHS overlap that is specific to case subjects.

(A)	Select “target area file”

We will restrict the regions to be analyzed to those shown by “HM_RHSsSharedBy005_st001_st002_st003_st004_st005.ps”. The regions are listed in HM_RHSsSharedBy005_st001_st002_st003_st004_st005.txt. Therefore, we use this file as the target area file.

Copy “HM_RHSsSharedBy005_st001_st002_st003_st004_st005.txt” from “ResultDirectory” to “TargetAreaFileDirectory”.

/home/myhomedirectory/Desktop/ResultDirectory/HM_RHSsSharedBy005_st001_st002_st003_st004_st005.txt

-> /home/myhomedirectory/Desktop/TargetAreaFileDirectory/HM_RHSsSharedBy005_st001_st002_st003_st004_st005.txt

(B)	Control

“/TutorialAndProgram/SampleDataSets/HapMap3JPNSynthetic” directory contains genotyping data files that were made from HapMap3JPT files, and each file give the same RHSs as the original data when analyzed by HMAnalysisProg. This is to provide sample datasets while avoiding the distribution of original data without permission. Files have already been copied to “ControlDirectory” at Step (I)-(B)-3.

(C)	Analysis file

Copy “/TutorialAndProgram/SampleAnalysisFile/Linux/CaseControlAnalysis/CaseControlAnalysisFile.txt” to “AnalysisFileDirectory”.

```
/TutorialAndProgram/SampleAnalysisFile/Linux/CaseControlAnalysis/CaseControlAnalysisFile.txt
   -> /home/myhomedirectory/Desktop/AnalysisFileDirectory/RHSCutoffAnalysisFile.txt
```

Open the file. **Change “myhomedirectory” to the name of your home directory.**

::patient pool

/home/myhomedirectory/Desktop/CaseDirectory/01.txt

/home/myhomedirectory/Desktop/CaseDirectory/02.txt

/home/myhomedirectory/Desktop/CaseDirectory/03.txt

/home/myhomedirectory/Desktop/CaseDirectory/04.txt

/home/myhomedirectory/Desktop/CaseDirectory/05.txt

/home/myhomedirectory/Desktop/CaseDirectory/06.txt

::control pool

/home/myhomedirectory/Desktop/ControlDirectory/01.txt

/home/myhomedirectory/Desktop/ControlDirectory/02.txt

/home/myhomedirectory/Desktop/ControlDirectory/03.txt

/home/myhomedirectory/Desktop/ControlDirectory/04.txt

/home/myhomedirectory/Desktop/ControlDirectory/05.txt

/home/myhomedirectory/Desktop/ControlDirectory/06.txt

/home/myhomedirectory/Desktop/ControlDirectory/07.txt

/home/myhomedirectory/Desktop/ControlDirectory/08.txt

/home/myhomedirectory/Desktop/ControlDirectory/09.txt

/home/myhomedirectory/Desktop/ControlDirectory/10.txt

/home/myhomedirectory/Desktop/ControlDirectory/11.txt

/home/myhomedirectory/Desktop/ControlDirectory/12.txt

/home/myhomedirectory/Desktop/ControlDirectory/13.txt

/home/myhomedirectory/Desktop/ControlDirectory/14.txt

/home/myhomedirectory/Desktop/ControlDirectory/15.txt

/home/myhomedirectory/Desktop/ControlDirectory/16.txt

/home/myhomedirectory/Desktop/ControlDirectory/17.txt

/home/myhomedirectory/Desktop/ControlDirectory/18.txt

/home/myhomedirectory/Desktop/ControlDirectory/19.txt

/home/myhomedirectory/Desktop/ControlDirectory/20.txt

/home/myhomedirectory/Desktop/ControlDirectory/21.txt

/home/myhomedirectory/Desktop/ControlDirectory/22.txt

/home/myhomedirectory/Desktop/ControlDirectory/23.txt

/home/myhomedirectory/Desktop/ControlDirectory/24.txt

/home/myhomedirectory/Desktop/ControlDirectory/25.txt

/home/myhomedirectory/Desktop/ControlDirectory/26.txt

/home/myhomedirectory/Desktop/ControlDirectory/27.txt

/home/myhomedirectory/Desktop/ControlDirectory/28.txt

/home/myhomedirectory/Desktop/ControlDirectory/29.txt

/home/myhomedirectory/Desktop/ControlDirectory/30.txt

/home/myhomedirectory/Desktop/ControlDirectory/31.txt

/home/myhomedirectory/Desktop/ControlDirectory/32.txt

/home/myhomedirectory/Desktop/ControlDirectory/33.txt

/home/myhomedirectory/Desktop/ControlDirectory/34.txt

/home/myhomedirectory/Desktop/ControlDirectory/35.txt

/home/myhomedirectory/Desktop/ControlDirectory/36.txt

/home/myhomedirectory/Desktop/ControlDirectory/37.txt

/home/myhomedirectory/Desktop/ControlDirectory/38.txt

/home/myhomedirectory/Desktop/ControlDirectory/39.txt

/home/myhomedirectory/Desktop/ControlDirectory/40.txt

/home/myhomedirectory/Desktop/ControlDirectory/41.txt

/home/myhomedirectory/Desktop/ControlDirectory/42.txt

/home/myhomedirectory/Desktop/ControlDirectory/43.txt

/home/myhomedirectory/Desktop/ControlDirectory/44.txt

/home/myhomedirectory/Desktop/ControlDirectory/45.txt

/home/myhomedirectory/Desktop/ControlDirectory/46.txt

/home/myhomedirectory/Desktop/ControlDirectory/47.txt

/home/myhomedirectory/Desktop/ControlDirectory/48.txt

/home/myhomedirectory/Desktop/ControlDirectory/49.txt

/home/myhomedirectory/Desktop/ControlDirectory/50.txt

/home/myhomedirectory/Desktop/ControlDirectory/51.txt

/home/myhomedirectory/Desktop/ControlDirectory/52.txt

/home/myhomedirectory/Desktop/ControlDirectory/53.txt

/home/myhomedirectory/Desktop/ControlDirectory/54.txt

/home/myhomedirectory/Desktop/ControlDirectory/55.txt

/home/myhomedirectory/Desktop/ControlDirectory/56.txt

/home/myhomedirectory/Desktop/ControlDirectory/57.txt

/home/myhomedirectory/Desktop/ControlDirectory/58.txt

/home/myhomedirectory/Desktop/ControlDirectory/59.txt

/home/myhomedirectory/Desktop/ControlDirectory/60.txt

/home/myhomedirectory/Desktop/ControlDirectory/61.txt

/home/myhomedirectory/Desktop/ControlDirectory/62.txt

/home/myhomedirectory/Desktop/ControlDirectory/63.txt

/home/myhomedirectory/Desktop/ControlDirectory/64.txt

/home/myhomedirectory/Desktop/ControlDirectory/65.txt

/home/myhomedirectory/Desktop/ControlDirectory/66.txt

/home/myhomedirectory/Desktop/ControlDirectory/67.txt

/home/myhomedirectory/Desktop/ControlDirectory/68.txt

/home/myhomedirectory/Desktop/ControlDirectory/69.txt

/home/myhomedirectory/Desktop/ControlDirectory/70.txt

/home/myhomedirectory/Desktop/ControlDirectory/71.txt

/home/myhomedirectory/Desktop/ControlDirectory/72.txt

/home/myhomedirectory/Desktop/ControlDirectory/73.txt

/home/myhomedirectory/Desktop/ControlDirectory/74.txt

/home/myhomedirectory/Desktop/ControlDirectory/75.txt

/home/myhomedirectory/Desktop/ControlDirectory/76.txt

/home/myhomedirectory/Desktop/ControlDirectory/77.txt

/home/myhomedirectory/Desktop/ControlDirectory/78.txt

/home/myhomedirectory/Desktop/ControlDirectory/79.txt

/home/myhomedirectory/Desktop/ControlDirectory/80.txt

/home/myhomedirectory/Desktop/ControlDirectory/81.txt

/home/myhomedirectory/Desktop/ControlDirectory/82.txt

/home/myhomedirectory/Desktop/ControlDirectory/83.txt

/home/myhomedirectory/Desktop/ControlDirectory/84.txt

/home/myhomedirectory/Desktop/ControlDirectory/85.txt

/home/myhomedirectory/Desktop/ControlDirectory/86.txt

::excluded area file

::target area file

/home/myhomedirectory/Desktop/TargetAreaFileDirectory/HM_RHSsSharedBy005_st001_st002_st003_st004_st005.txt

::annotation file

/home/myhomedirectory/Desktop/AnnotationFileDirectory/annotationJapanese1000k.txt

::output directory

/home/myhomedirectory/Desktop/ResultDirectory

::suspected genotyping error rate

0.003

CaseControlAnalysisFile.txt

(D)	Launch the program. The analysis is limited to the regions as described in the target area file.

```
>/home/myhomedirectory/Desktop/HMAnalysisProgramDirectory/HMAnalysisProg
  /home/myhomedirectory/Desktop/AnalysisFileDirectory/CaseControlAnalysisFile.txt
```

If you cannot launch program because of “permission denied” message, change the permission of the program using chmod.

chmod +x /home/myhomedirectory/Desktop/HMAnalysisProgramDirectory/HMAnalysisProg

Input 0.6 when the program asks you the RHS cutoff.

Input RHS cutoff in cM: 0.6

(E)	Result

1a	HM_P_Value_PatientPool_vs_ControlPool.ps

A PostScript file that graphically shows –log_10_(P) value, as in the Figure 5c.

1b	HM_P_Value_PatientPool_vs_ControlPool.txt

A text file that summarizes –log_10_(P) for each region of the autosomes.

```
1	16676928	29.938894	rs156847	0.320975	5	67	start
1	16687581	29.957878	rs156851	0.320975	5	67	end
1	16701089	29.981950	rs696605	0.004571	5	84	start
1	16704450	29.987940	rs7537985	0.004571	5	84	end
1	16718622	30.013196	rs156913	0.000019	5	86	start
1	16792858	30.145490	rs9724879	0.000019	5	86	end
1	16795674	30.150509	rs675952	0.007315	6	86	start
1	17155012	30.790878	rs2143808	0.007315	6	86	end
```

The columns indicate chromosome number, nucleotide position, genetic map position in centimorgans from the terminal of the short arm of the chromosome, dbSNP rsID, -log_10_(P) value, number of subjects in case sharing RHS, number of subjects in control sharing RHS, and the label showing the start and the end of the region.

**Appendix**

(A)	File format

1.	Genotyping data

Genotyping data is a text file with a format as shown below.

```
rs2887286	AB
rs1496555	AB
rs41477744	BB
rs3890745	AB
rs10492936	AA
rs10489588	AB
rs2376495	BB
rs4648462	AA
….	….
```

The first column is the dbSNP rsID, which is separated by a tab code from the second column that is genotype. The genotype is presented by “AA”, “AB”, “BB” or “NoCall”. Affymetrix and Illumina have different convention of nomenclature for assigning “AA”, “AB” or “BB”. Therefore, data obtained by platforms from different manufacturer cannot be used interchangeably.

2.	Annotation file

Annotation file is a text file with a format as shown below. Annotation file for Affymetrix can be obtained from http://www.hhanalysis.com. Annotation file for Illumina have not got permission from Illumina to make public on the web. For Annotation file for Illumina platform should thus be directly requested to hagiwark@mac.com.

```
1	742429	0.144444	rs3094315	0.830754	0
1	767376	0.000000	rs6672353	0.858668	1
1	769185	0.866667	rs4040617	0.860693	2
1	775852	0.397727	rs2980300	0.868153	3
…
```

Each line describes information for a single SNP; chromosome number, nucleotide position, genetic map position in centimorgans from the terminal of the short arm of the chromosome, dbSNP rsID, the frequency of “A” allele, and the serial number.

(B)	Compilation (Optional)

The package is consisted of two programs, RHSCutoffDeterminationProg and HMAnalysisProg. The binary files are included. However, in case you want to compile the program from the source codes, a makefile for GNU C compiler (http:gcc.gnu.org/) is included with each source file. Copy the source file desktop. Change the current directory to the source file directory, and compile using the makefile.

RHSCutoffDeterminationProg

```
/TutorialAndProgram/SourceCode ->
	/home/myhomedirectory/Desktop/SourceCode

cd /home/myhomedirectory/Desktop/SourceForRHSCutoffDeterminationProg

makefile –f /home/myhomedirectory/Desktop/SourceCode/MakeFileRHSCutoffDeterminationProg
```

HMAnalysisProg

```
/TutorialAndProgram/SourceCode ->
	/home/myhomedirectory/Desktop/SourceCode

cd /home/myhomedirectory/Desktop/SourceForHMAnalysisProg

makefile –f /home/myhomedirectory/Desktop/SourceCode/MakeFileHMAnalysisProg
```
